# Integrated seroprevalence-based assessment of *Wuchereria bancrofti* and *Onchocerca volvulus* in two lymphatic filariasis evaluation units of Mali with the SD Bioline Onchocerciasis/LF IgG4 Rapid Test

**DOI:** 10.1371/journal.pntd.0007064

**Published:** 2019-01-30

**Authors:** Housseini Dolo, Yaya Ibrahim Coulibaly, Benoit Dembele, Boubacar Guindo, Siaka Yamoussa Coulibaly, Ilo Dicko, Salif Seriba Doumbia, Massitan Dembele, Mamadou Oumar Traore, Seydou Goita, Mamadou Dolo, Lamine Soumaoro, Michel Emmanuel Coulibaly, Abdallah Amadou Diallo, Dansine Diarra, Yaobi Zhang, Robert Colebunders, Thomas B. Nutman

**Affiliations:** 1 Filariasis Unit, International Center of Excellence in Research, Faculty of Medicine and Odontostomatology, Point G, Bamako, Mali; 2 Global Health Institute, Faculty of Medicine & Health Sciences, University of Antwerp, Antwerp, Belgium; 3 Centre National d’Appui à la Lutte contre la Maladie, Bamako, Mali; 4 Neglected Tropical Diseases Control Programme, Helen Keller Iinternational Bamako, Mali; 5 Programme National d’Elimination de la Filariose Lymphatique, Bamako, Mali; 6 Programme National de Lutte contre l’Onchocercose, Bamako, Mali; 7 Institut National de Recherche en Santé Publique, Bamako, Mali; 8 Faculty of Geography and History, Bamako, Mali; 9 Helen Keller International, Regional Office for Africa, Dakar, Senegal; 10 Laboratory of Parasitic Diseases, National Institute of Allergy and Infectious Diseases, National Institutes of Health, Bethesda, United States of America; University of Buea, CAMEROON

## Abstract

**Background:**

Mali has become increasingly interested in the evaluation of transmission of both *Wuchereria bancrofti* and *Onchocerca volvulus* as prevalences of both infections move toward their respective elimination targets. The SD Bioline Onchocerciasis/LF IgG4 Rapid Test was used in 2 evaluation units (EU) to assess its performance as an integrated surveillance tool for elimination of lymphatic filariasis (LF) and onchocerciasis.

**Methodology/Principal findings:**

A cross sectional survey with SD Bioline Onchocerciasis/LF IgG4 Rapid Test was piggy-backed onto a transmission assessment survey (TAS) (using the immunochromatographic card test (ICT) Binax Filariasis Now test for filarial adult circulating antigen (CFA) detection) for LF in Mali among 6–7 year old children in 2016 as part of the TAS in two EUs namely Kadiolo-Kolondieba in the region of Sikasso and Bafoulabe -Kita-Oussoubidiagna-Yelimane in the region of Kayes.

In the EU of Kadiolo- Kolondieba, of the 1,625 children tested, the overall prevalence of *W*. *bancrofti* CFA was 0.62% (10/1,625) [CI = 0.31–1.09]; while that of IgG4 to Wb123 was 0.19% (3/1,600) [CI = 0.04–0.50]. The number of positives tested with the two tests were statistically comparable (p = 0.09). In the EU of Bafoulabe-Kita-Oussoubidiagna-Yelimane, an overall prevalence of *W*. *bancrofti* CFA was 0% (0/1,700) and that of Wb123 IgG4 antibody was 0.06% (1/1,700), with no statistically significant difference between the two rates (p = 0.99).

In the EU of Kadiolo- Kolondieba, the prevalence of Ov16-specific IgG4 was 0.19% (3/1,600) [CI = 0.04–0.50]. All 3 positives were in the previously *O*. *volvulus*-hyperendemic district of Kolondieba. In the EU of Bafoulabe-Kita-Oussoubidiagna-Yelimane, an overall prevalence of Ov16-specific IgG4 was 0.18% (3/1,700) [CI = 0.04–0.47]. These 3 Ov16 IgG4 positives were from previously *O*.*volvulus*-mesoendemic district of Kita.

**Conclusions/Significance:**

The SD Bioline Onchocerciasis/LF IgG4 Rapid test appears to be a good tool for integrated exposure measures of LF and onchocerciasis in co-endemic areas.

## Author summary

After years of mass drug administration for lymphatic filariasis and onchocerciasis, many co-endemic countries need to demonstrate the interruption of transmission of these parasitic infections. This interruption of transmission is the key to stopping mass drug administration. One of the major challenges facing national programs is a reliable point of care diagnostic tool capable of detecting recent infection in children. To this end, the SD Bioline Onchocerciasis / LF IgG4 Rapid Test was used to evaluate the transmission interruption of lymphatic filariasis and onchocerciasis in previously endemic regions of Mali. This rapid diagnostic tool demonstrated good performance in 6 of districts in 2 “so-called’ evaluation units. There was very good overlap of the serological results obtained by the rapid diagnostic test (RDT) and previous endemicity profile of the different study districts for the two infections. We would argue that this RDT could be a single integrated approach for assessing transmission in areas where lymphatic filariasis and onchocerciasis are/were co-endemic.

## Introduction

Approaches to onchocerciasis control and lymphatic filariasis (LF) elimination have proceeded along parallel but independent courses in Mali. For LF, annual mass drug administration (MDA) with albendazole and ivermectin began in 2004 and continued for 5 to 9 consecutive MDA campaigns. Onchocerciasis was originally highly endemic in five regions of Mali (Kayes, Koulikoro, Sikasso, Segou and Mopti [1]; the eastern most regions (Koulikoro rive droite, Sikasso, Ségou and Mopti) were included in the original onchocerciasis control programme (OCP) based on vector control with larvicides [2]. The western parts of the endemic regions (Kayes and Koulikoro rive gauche) were part of the western extension of OCP in which ivermectin administration was used in MDA compaigns and later community directed therapeutic intervention (CDTi) under the umbrella of African Programme for Onchocerciasis Control (APOC) using community drug distributors (CDDs).

LF and onchocerciasis are overlapping in many districts of Mali [1]. Both the LF and onchocerciasis control programs were implemented based on precontrol mapping data for each of diseases [1]. For meso- and hyper-endemic onchocerciasis, the use of skin snips and eye examination for mapping demonstrated that 5 of the 8 administrative regions of Mali were endemic for *O*. *volvulus* [1,3]; whereas all of the 8 administrative units (encompassing 75 health districts) were endemic for LF based on a 2004 survey using the immunochromatographic test (ICT) known commercially as the Binax Filariasis Now test (Alere, Portland, ME) where circulating filarial antigen (CFA) prevalences were greater than 1% [1,4].

In 2005, the results of longitudinal studies from 3 formerly *O*. *volvulus* hyperendemic foci in Mali and Senegal provided evidence that onchocerciasis elimination could be achieved based only on mass drug adminstration of ivermectin [5]. The evaluations used skin snips for the detection of microfilaridermia and blackfly dissection [5,6]. At the same time, when LF was mapped and reported to be endemic throughout the country, all the districts in Mali were treated using MDA (ivermectin with albendazole) for LF.

LF transmission assessment surveys (TAS) as recommended by World Health Organization (WHO) were initiated in 2012 and are currently being performed in 22 evaluation units (EU). An EU includes one or several endemic districts based on geographic location, treatment coverage and population size [7].

The Binax Filariasis Now ICT cards and more recently the Filariasis Test Strip (FTS) [8] have been used for LF mapping and for the TAS, but these tests may have their limitations because of their slow kinetics of disappearance and their potential cross-reaction in cases of *Loa loa* infection, a filarial parasite that is absent in Mali [9]. For onchocericasis, post-treatment surveillance based on positivity in children with Ov16-based immunoassays is the current gold standard, but the challenge remains in the definition of prevalence cutoffs using the various forms of the Ov16 ELISA [10] or the SD Bioline’s Ov16-containing RDTs [11].

Initially, *O*. *volvulus* infection mapping in Mali was conducted using skin snip and eye examination that left many hypo-endemic areas excluded from the various control programs and from further consideration for CDTi. As a consequence of “redistricting” in 2016, the number of onchocerciasis-endemic districts increased from 17 to 34. Among these 34, only 2 have stopped CDTi. Hence, re-mapping is needed in many potentially *O*. *volvulus* -endemic areas. Mass drug adminstration of ivermectin is still ongoing in 20 districts, and 12 are under surveillance. These 12 districts (under surveillance for onchocerciasis) had previously been part of the OCP vector control program; however, they have received albendazole and ivermectin for LF for at least 5 MDA rounds. Beyond these 34 districts, there may be a need for re-mapping potentially *O*. *volvulus*-hypo-endemic regions if elimination goals are to be achieved in the near future [12].

In this study, the SD Bioline Onchocerciasis/LF IgG4 Rapid Test was used concurrently with the ICT in two LF evaluation units (EU) in Mali consisting of districts with different endemicity levels for LF and onchocerciasis prior to MDA/CDTi to assess its performance as an integrated surveillance tool for LF and onchocerciasis elimination.

## Methods

### Ethics statement

As this work was conducted as part of the National Neglected Tropical Diseases control program activity, it was deemed exempt from IRB approval by the ethical committee of the University of Science, Techniques and Technologies of Bamako. However, a protocol related to the evalution of SD Bioline Onchocerciasis/LF IgG4 Rapid Test in Mali was approved by the ethical committee (2017/199/CE/FMPOS). Participation in this study was entirely voluntary. The study was clearly explained to the community leaders and health authorities and their permission obtained before any activities were undertaken. Oral consent was obtained from the legal guardians of all the 6- to 7-year old children due to low literacy level in communities.

### Study area

In the Sikasso region, the study was conducted in the EU of the district of Kadiolo and Kolondièba. In this EU, the district of Kadiolo had been endemic for *W*. *bancrofti* (24.4% pre-control) but hypoendemic for *O*. *volvulus* (20% pre-control), while the district of Kolondieba had been co-endemic for these two parasites (37% and 60% pre-control prevalences for LF and onchocerciasis respectively) [1,13] (Table 1).

**Table 1:** Lymphatic filariasis and onchocerciasis pre-control endemicity and current status of mass drug distribution per district.

Evaluation Unit	Districts	Endemicity		Precontrol prevalence Lymphatic Filariasis	Number Ivermectin/ Albendazole MDA	Last year Lymphatic Filariasis MDA	Pre-control prevalence onchocerciasis	Number ivermectin CDTi	Last year CDTi
		LF	Onchocerciasis						
Kadiolo -Kolondieba	Kadiolo	Endemic	Hypoendemic	24.4%	8	2016	20%	NA	NA
	Kolondieba	Endemic	Hyperendemic	37%	9	2016	60%	25	Ongoing
Bafoulabe -Kita-Oussoubidiagna-Yelimane	Bafoulabe	Endemic	Mesoendemic	9.6%	7	2016	42%	25	Ongoing
	Kita	Endemic	Mesoendemic	9.6%	6	2016	40%	24	Ongoing
	Oussoubidiagna	Endemic	Hyperendemic	9.6%	6	2016	60%	25	Ongoing
	Yelimane	Endemic	Hypoendemic	9.6%	7	2016	33%	NA	NA

MDA = mass drug administration, LF = lymphatic Filariasis, Prev = prevalence %, CDTi = community directed treatment with ivermectin, NA = not applicable

In Kayes region, the entire EU of the district of Bafoulabe, Kita, Oussoubidiagna and Yelimane was known to be endemic for LF (with 9.6% pre-control prevalence in each district), but *O*. *volvulus* was found to be endemic in certain districts with pre-control prevalences as follows: Kita (mesoendemic-40%), Bafoulabe (mesoendemic—42%), Oussoubidiagna (hyperendemic-60%), Yelimane (hypoendemic—33%) (Table 1). In all these districts, 2016 was the last year of ivermectin and albendazole distribution for LF although mass drug adminstration of ivermectin continues for onchocerciasis [1,13].

### Study design

The present study was piggy-backed onto TAS surveys (using the Binax Filariasis Now test for filarial adult circulating antigen detection) for LF across 2 EUs in Mali to demonstrate the utility of the SD Bioline Onchocerciasis/LF IgG4 Rapid Test for integrated assessment of *W*. *bancrofti* and *O*. *volvulus* transmission [14].

### Sampling and participants

The sample size builder (SSB) was used to automate the calculations for determining appropriate survey strategy and sample size calculations based on TAS sampling strategy. The design of the surveys is flexible in order to best fit the local situation and depends upon factors such as the primary school enrolment rate, the populations size, the number of schools or enumeration areas, and the cost of different survey methods [7]. For this current study community based survey was conducted due to low school enrollment rate.

The number of villages and the number of households included in the study were determined by the SSB [7]. Children of 6 and 7 year old within the randomly selected households made the sample. The minimum size required for the TAS were 1,556 and 1,692 children 6–7 year old (according to the SSB) respectively for EUs of Kadiolo-Kolondieba and Bafoulabe -Kita-Oussoubidiagna-Yelimane. The estimated total population of 6 to 7 year old children were 37,620 and 71,152 respectively in the EU of Kadiolo- Kolondieba and Bafoulabe-Kita-Oussoubidiagna-Yelimane (Table 2).

**Table 2:** Study area description per evaluation unit and details on number of villages and mean number of expected children.

Evaluation Unit	Districts per evaluation unit	Number of villages per district	Number of villages per evaluation unit	Number children 6–7 year of age per evaluation unit	Mean Number of expected children 6–7 year of age per village
Kadiolo -Kolondieba	Kadiolo	11	322	37,620	117
	Kolondieba	19			
	Total	30			
Bafoulabe -Kita-Oussoubidiagna-Yelimane	Bafoulabe	8	665	71,152	107
	Kita	15			
	Oussoubidiagna	5			
	Yelimane	3			
	Total	31			

A multistage sampling technique was used for the sampling. In each EU, the villages and the backup villages were randomly selected using the SSB tool. The backup villages were chosen in addition to 30 villages that made the cluster for an EU. In the one case where the selected village was inaccessible or refused to participate in the survey, a back up village was used to replace it. At the village level, the list of the households was made available, and two tables of random numbers generated from computer using the SSB were also used to select randomly the households to be included in the study. In each selected household, all the children 6–7 years of age were included in the study. A household was defined as a group of persons living in the same house and sharing the same food.

### Circulating filarial antigen and antibody measurement

Children aged 6-and 7-year-old were tested using Binax Filariasis Now test and retested ~ 6 months later with the SD Bioline Onchocerciasis/LF IgG4 Rapid Test in the EU of Kadiolo–Kolondieba at the point of care (POC) (Table 2). In the EU of Bafoulabe -Kita-Oussoubidiagna-Yelimane, dried blood spots (DBS) were collected and cryopreserved at the time of the TAS as the SD Bioline Onchocerciasis/LF IgG4 Rapid Test were not available. All tests for CFA were performed at the POC using the Binax Filariasis Now test (Alere, Portland, ME), using the manufacturer’s instructions. In the EU of Kadiolo–Kolondieba, the SD Bioline Onchocerciasis/LF IgG4 Rapid Test was used at the POC again exactly as recommended by the manufacturer (SD Diagnostics, Korea). For the dried blood spots used for antibody assessments in the EU of Bafoulabe-Kita-Oussoubidiagna-Yelimane, the blood was collected on filter paper spots (TropBio, Townsville, Australia) and cryopreserved in liquid nitrogen dry shippers in the field before being transported to the Filariasis Research Unit laboratory in Bamako the capital city of Mali. The DBS were then stored at– 80°C prior to elution as described in Kamgno et al, [15] and tested using the SD Bioline Onchocerciasis/LF IgG4 Rapid Test. The SD Bioline Onchocerciasis/LF IgG4 Rapid Test measures simultaneously the presence of IgG4 antibodies to Wb123 and Ov16.

### Statistical analysis

All data analyses were performed using SPSS Version 24 (Statistical package for Social Sciences) and used the 5% level of significance. The Fisher's exact test was used when appropriate and the Clopper-Pearson 95% confidence interval around the prevalences were used for statistical comparisons. The geographic coordinates were measured for each village visited during the survey using mobile phones and thereafter used to map the ICT positive individuals as well as those positive for IgG4 antibodies against Wb123 and Ov16.

## Results

In the EU of Kadiolo- Kolondieba, 1,625 children aged of 6–7 year old were tested at the point of care (POC) using the Binax Filariasis Now ICT cards; whereas 1,600/1,625 (98.5%) were tested using the POC SD Bioline Onchocerciasis/LF IgG4 Rapid Test. In the EU of Bafoulabe-Kita-Oussoubidiagna-Yelimane, 1,700 children aged of 6–7 year old were tested at the POC using Binax Filariasis Now test and retested using elutions of DBS on the SD Bioline Onchocerciasis/LF IgG4 Rapid Test (Table 3).

**Table 3:** Study sites and population tested.

Evaluation Unit	Districts	Number villages /district	Number tested Binax Filariasis Now test	Endemicity level* Onchocerciasis	Number tested Ov16/WB123 RDT
Kadiolo -Kolondieba	Kadiolo	11	581	Hypoendemic	502
	Kolondieba	19	1,044	Hyperendemic	1,098
	Total	30	1,625	NA	1,600
Bafoulabe -Kita-Oussoubidiagna-Yelimane	Bafoulabe	8	461	Mesoendemic	461
	Kita	15	713	Mesoendemic	713
	Oussoubidiagna	5	348	Hyperendemic	348
	Yelimane	3	178	Hypoendemic	178
	Total	31	1,700	NA	1,700

*All districts previously LF endemic (>1% prevalence); Binax Filariasis Now test = Immunochromatographic cards test, NA = not applicable, LF = lymphatic filariasis; RDT = rapid diagnostic test

### CFA and Wb123 IgG4 antibody prevalence

In the EU of Kadiolo- Kolondieba, an overall prevalence of *W*. *bancrofti* infection based on CFA was found to be 0.62% [95% CI = 0.31–1.09]. When assessed at the district level, the CFA prevalence was 0.69% [95% CI = 0.21–1.65] in Kadiolo and 0.57% [95% CI = 0.23–1.19] in Kolondieba. In the same EU, the overall prevalence of Wb123 IgG4 was 0.19% [95% CI = 0.04–0.50]; when assessed per district, the prevalence of IgG4 to Wb123 was 0.20% [95% CI = 0.0–0.97] in Kadiolo and 0.18% [95% CI = 0.03–0.60] in Kolondieba (Table 4). The prevalences obtained using either of the two tests were statistically comparable (p = 0.99). In the EU of Bafoulabe-Kita-Oussoubidiagna-Yelimane, an overall prevalence of *W*. *bancrofti* infection based on CFA in chidren was 0% (0/1,700). For the Wb123 IgG4 antibody, only 1/1,700 (0.06% [95% CI = 0.0–0.28]) was found. This single positive was reported in the district of Bafoulabe; thus the local prevalence of Wb123 IgG4 was 0.21% [95% CI = 0.01–1.06] (Table 4). There were no differences between the CFA positive cases and those positive for Wb123 (p = 0.99).

**Table 4:** Circulating filarial antigen and *Wuchereria bancrofti* seroprevalence per district and evaluation unit.

Evaluation Unit	Districts	Number tested Binax Filariasis Now test	Number Positive Binax Filariasis Now test	% Pos Binax Filariasis Now test, [95% CI]	Number tested Biplex	Number Positive Wb123	% Positive Wb123, [95% CI]
Kadiolo -Kolondieba	Kadiolo	581	4	0.69 [0.21–1.65]	502	1	0.20 [0.00–0.97]
	Kolondieba	1,044	6	0.57 [0.23–1.19]	1,098	2	0.18 [0.03–0.60]
	Total	1,625	10	0.62 [0.31–1.09]	1,600	3	0.19 [0.04–0.50]
Bafoulabe -Kita-Oussoubidiagna-Yelimane	Bafoulabe	461	0	0 [0.0–0.64]	461	1	0.21 [0.01–1.06]
	Kita	713	0	0 [0.0–0.41]	713	0	0[0.0–0.42]
	Oussoubidiagna	348	0	0 [0.0–0.85]	348	0	0 [0.0–85]
	Yelimane	178	0	0 [0.0–1.66]	178	0	0 [0.0–1.66]
	Total	1,700	0	0 [0.0–0.17]	1,700	1	0.06 [0.0–0.28]

% = percentage, Binax Filariasis Now test = immunochromatographique card test, CI = confidence interval, Pos = positive, Wb123: *Wuchereria bancrofti* specific antibodies 123

### Ov16 IgG4 antibody prevalence

In the EU of Kadiolo–Kolondieba, 3/1,600 children were positive for Ov16-specific IgG4 with an overall antibody prevalence of 0.19% [95% CI = 0.04–0.50] being found. All 3 positives were in the district of Kolondieba, leading to a local prevalence of 0.27% [95% CI = 0.06–0.74] in this previously *O*. *volvulus*-hyperendemic district (Table 5). It should be noted that 0/502 children in Kadiolo (an *O*.*volvulus*-hypoendemic region) were IgG4 positive to Ov16, but the upper 95% confidence level around this 0% prevalence was 0.59%.There was no statistical difference between the number positive (0/502) in Kadiolo, an *O*. *volvulus*-hypoendemic district and Kolondieba a previously *O*. *volvulus*-hyperendemic district (3/1,095) (p = 0.55). In the EU of Bafoulabe-Kita-Oussoubidiagna-Yelimane, an overall prevalence of Ov16-specific IgG4 of 0.18% [95% CI = 0.04–0.47] was recorded. These 3 individual Ov16 IgG4 positives were in the district of Kita among the 713 children tested, giving a prevalence of 0.42% for this district [95% CI = 0.10–1.14] (Table 5).

**Table 5:** *Onchocerca volvulus* seroprevalence per district and evaluation unit according to pre-control endemicity.

Evaluation Unit	Districts	*O*. *volvulus* endemicity	Number tested Biplex	Number Positive Ov16	% Positive Ov16 [95% CI]
Kadiolo -Kolondieba	Kadiolo	Hypoendemic	502	0	0 [0.0–0.59]
	Kolondieba	Hyperendemic	1,098	3	0.27 [0.06–0.74]
	Total	NA	1,600	3	0.19 [0.04–0.50]
Bafoulabe -Kita-Oussoubidiagna-Yelimane	Bafoulabe	Mesoendemic	461	0	0 [0.0–0.64]
	Kita	Mesoendemic	713	3	0.42 [0.10–1.14]
	Oussoubidiagna	Hyperendemic	348	0	0 [0.0–0.85]
	Yelimane	Hypoendemic	178	0	0 [0.0–1.66]
	Total	Not applicable	1,700	3	0.18 [0.04–0.47]

Ov16 = *Onchocerca volvulus* specific antibodies 16, CI = confidence interval, % = percentage

Again it should be noted that in the *O*. *volvulus*-hypoendemic district (Yelimane), although there were no Ov16 positive children, the upper limit of the 95% CI exceeded 1%, suggesting that cut off point of antibody positivity for children should be raised above the 0.1% threshold. The comparison of positive cases of Ov16 between the previous *O*. *volvulus*-hypoendemic district (Yelimane) and the previous *O*. *volvulus*-mesoendemic district with 3 positives cases was similar (p = 0.99).

### LF and onchocerciais geographic distribution

**Fig 1:**
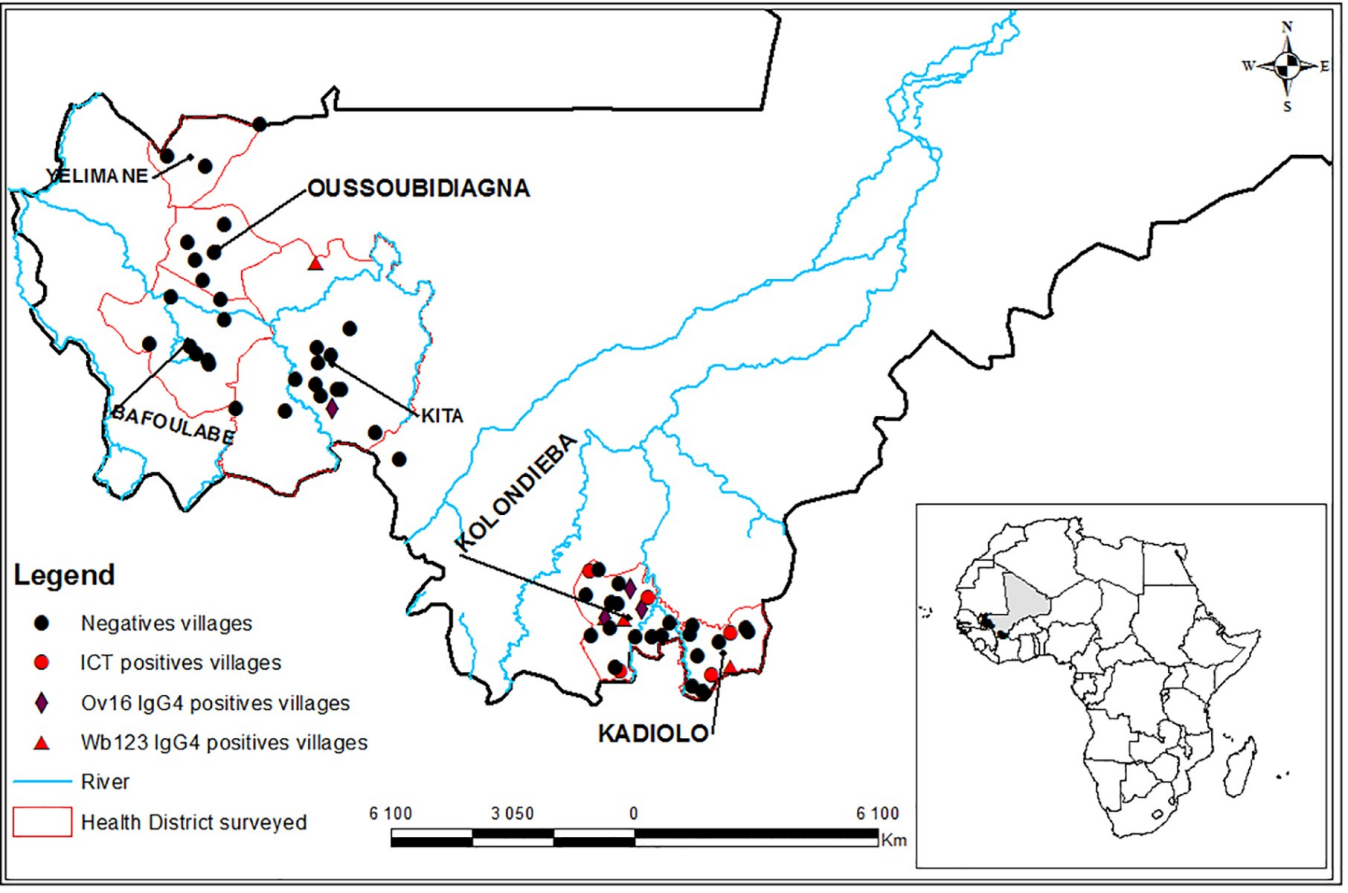
Distribution of circulating filarial antigen (orange dots) IgG4 antibodies to Wb123 (red dots) and to Ov16 (blue dots) in the evaluation units of Kadiolo–Kolondieba and Bafoulabe -Kita-Oussoubidiagna-Yelimane. Source: figure created by Dansine Diarra (geographer) specifically for this article. The authors retain copyright and authorize its distribution open access.

CFA positive children were found primarily in the EU of Kadiolo-Kolondieba with 10 CFA positives detected using Binax Filariasis Now test in five different villages (Fig 1). No CFA carriers were found in the 31 villages of the Bafoulabe-Kita-Oussoubidiagna-Yelimane EU, but one child was found positive for Wb123 IgG4 in the district of Bafoulabe (Fig 1). In the EU of Kadiolo Kolondieba, 3 positive Wb123 IgG4 children were observed in three different villages. In the same EU, 3 Ov16 IgG4 positive children were found in 3 different villages (Fig 1). In the EU of Bafoulabe-Kita-Oussoubidiagna-Yelimane, 3 Ov16 IgG4 positive cases were detected in a single village of Kita district previously mesoendemic at the pre-control (Fig 1).

Overall, single Ov16 or Wb123 IgG4-positive children were spread across 5 different villages of the 30 villages screened in Kadiolo–Kolondieba EU (Fig 1).

## Discussion

Mali has become increasingly interested in the evaluation of transmission of both *O*. *volvulus* and *W*. *bancrofti* as prevalences of both infections move toward their respective elimination targets. This reduction is due to the fact that the national lymphatic filariasis elimination program, since its creation, has distributed the required number of mass treatment campaigns with at least 5 consecutive rounds of albendazole and ivermectin treatment; thus TAS is ongoing in the different EUs [1]. With regard to onchocerciasis, after periods of vector control, and ivermectin mass drug administration lasting more than 24 years in previously hyper- and meso-endemic foci, the program needs to fulfill the criteria for the cessation of ivermectin distribution [1]. Additionally, elimination goals likely were reached in most endemic foci by 2006 [6]. In 2016, as part of the TAS for LF using the Binax Filariasis Now test by the national LF elimination program in two EUs, namely Kadiolo-Kolondieba in the region of Sikasso and Bafoulabe-Kita-Oussoubidiagna-Yelimane in the region of Kayes, the SD Bioline Onchocerciasis/LF IgG4 Rapid Test was used to assess its performance as an integrated surveillance tool [14] to inform each of the two national elimination programs involved in LF and onchocericiasis elimination respectively.

For the assessment of *W*. *bancrofti* transmission, in the EU of Kadiolo- Kolondieba, *W*. *bancrofti* circulating filarial antigen (CFA) prevalence was 0.62% [95% CI = 0.31–1.09] while the seroprevalence (antibodies) targeting the same parasite was 0.19% [95% CI = 0.04–0.50]. In the EU of Bafoulabe-Kita-Oussoubidiagna-Yelimane, the CFA prevalence was 0% [95% CI = 0.0–0.17] while the antibody seroprevalence was 0.06% [95% CI = 0.0–0.28]. The prevalences of LF infection measured using these two tests were comparable and with all the tests, the upper bounds did not reach the cutoff point of 2% (Table 4).

Recent studies suggested that the Binax Filariasis Now test may overestimate the *W*. *bancrofti* infection prevalence [16,17]. Given that the specificity of the tests for CFA may be lower than previously thought in Loa-endemic areas [18], given that antibodies to Wb123 appear earlier than CFA in longitudinally followed children [19] and given the excellent superimposition of *W*. *bancrofti* prevalence results using either antibody (Wb123) or antigen (CFA) testing, we suggest that the measurement of IgG4 to Wb123 might be preferable in decisions to stop MDA or in TAS following cessation of MDA as it allows the measurement of recent exposure. The challenge remains, however, to determine an acceptable prevalence threshold that would guarantee interruption of *W*. *bancrofti* transmission particularly since vector monitoring is not part of most LF elimination programs. In the Gambia, the recent use of the Wb123 IgG4 test suggests that this serological tool could be used to decide to stop MDA [20]. Therefore, maybe the SD Bioline Onchocerciasis/LF IgG4 Rapid Test could be utilized for transmission assessment and as a decision tool for MDA cessation, should modelling studies help to corroborate these empiric findings.

Despite the application of the LF strategy for sampling of *O*. *volvulus* (not the recommended target age group less than 10 year old and sample size of 3,000 children) assessment, the level of exposure to *O*. *volvulus*, as determined by the detection of the IgG4 antibodies to Ov16, the current prevalence estimates overlaid quite nicely with previous epidemiological profiles of the different study districts (Tables 1,3 and 5). All Ov16 antibody positive subjects were reported in the previously known onchocerciasis meso- and/or hyperendemic districts [6]. Our results show that *O*. *volvulus* transmission level was above the current elimination threshold (0.1%) among the 6–7 year old, though the utility of this 0.1% target as a threshold is currently under debate [11,21]. The results of this study suggest that many of the hypoendemic areas are not in need of ivermectin distribution [22]. However, the sample size used in our study was calculated for LF transmission assessment and not for the evaluation of *O*. *volvulus* transmission (typically 3,000 children per transmission focus) [21].

Within the context of onchocerciasis elimination, new data are needed to re-categorize *O*. *volvulus* transmission potentials in subregions of countries such as Mali where many areas have undergone more than 24 years of CDTi with no or few epidemiological assessments [1]. Moreover, entomologic (blackfly) data are needed to confirm our findings that onchocerciasis transmission has been interrupted in these previously endemic district before to decide about CDTi cessation as suggested in a previous study [11]. The serological profiles reported here are similar to what was observed in southern Mexico where a decision to interrupt CDTi was taken [23].

Overall, the SD Bioline Onchocerciasis/LF IgG4 Rapid Test Wb123/Ov16 test, has 84% sensitivity and 98–99% specificity [24]. As no antibody-based test is likely to have specificities of >99% (meaning 1% false positive rates) the current threshold of elimination of onchocerciasis based on Ov16-specific antibodies (by whatever test available) must be increased and also results must be underscored by transmission assessment in pooled blackfly populations [25].

Based on the present study using the SD Bioline Onchocerciasis/LF IgG4 Rapid Test in previously LF- and onchocerciasis co-endemic regions in Mali, this POC test appears to be a good tool for integrated measurement of exposure to *W*. *bancrofti* and *O*. *volvulus* in children, with the potential to be used as a surrogate for interruption of transmission in a given focus as well as in post-MDA impact mesures in local populations in countries such as Mali. These data suggest that there is likely little or no transmission of either *O*. *volvulus* or *W*. *bancrofti*. Moreover, given the upper limits of the CI for Ov16 IgG4 in an *O*. *volvulus*-hypo-endemic region (and the fact that it does not differ from the hyper- and meso-endemic regions) a 0.1% threshold based on Ov-16-based IgG4 immunoassays may certainly be too conservative. Obviously, additional data underlying the utility of this POC test will be necessary if the elimination goals for both onchocerciasis and LF are to be achieved.
